# Cortical Contributions to Higher-Order Conditioning: A Review of Retrosplenial Cortex Function

**DOI:** 10.3389/fnbeh.2021.682426

**Published:** 2021-05-19

**Authors:** Danielle I. Fournier, Han Yin Cheng, Siobhan Robinson, Travis P. Todd

**Affiliations:** ^1^Department of Psychological and Brain Sciences, Dartmouth College, Hanover, NH, United States; ^2^Program in Neuroscience, Psychology Department, Hamilton College, Clinton, NY, United States

**Keywords:** higher-order conditioning, sensory preconditioning, second-order conditioning, retrosplenial cortex, associative learning

## Abstract

In higher-order conditioning paradigms, such as sensory preconditioning or second-order conditioning, discrete (e.g., phasic) or contextual (e.g., static) stimuli can gain the ability to elicit learned responses despite never being directly paired with reinforcement. The purpose of this mini-review is to examine the neuroanatomical basis of high-order conditioning, by selectively reviewing research that has examined the role of the retrosplenial cortex (RSC) in sensory preconditioning and second-order conditioning. For both forms of higher-order conditioning, we first discuss the types of associations that may occur and then review findings from RSC lesion/inactivation experiments. These experiments demonstrate a role for the RSC in sensory preconditioning, suggesting that this cortical region might contribute to higher-order conditioning via the encoding of neutral stimulus-stimulus associations. In addition, we address knowledge gaps, avenues for future research, and consider the contribution of the RSC to higher-order conditioning in relation to related brain structures.

## Introduction

Associative learning is one process by which animal behavior can be modified based on experience. One example of this is Pavlovian conditioning, in which animals learn predictive relationships between stimuli (Pavlov, 1927). In *first-order* conditioning, an excitatory association is formed between a conditioned stimulus (CS) and an unconditioned stimulus (US) that are directly paired together, if the CS provides predictive information about the US (Rescorla, 1972). Through these direct pairings, the CS will acquire the ability to elicit a conditioned response (CR). Stimuli can also acquire the ability to elicit CRs through *higher-order* conditioning, in which the CS is never directly paired with the US. Higher-order learning is critical for survival and likely contributes to a wide range of adaptive behaviors (Gewirtz and Davis, 2000), but may also contribute to the development and maintenance of psychiatric disorders, such as post-traumatic stress disorder (PTSD; Wessa and Flor, 2007).

**FIGURE 1:**
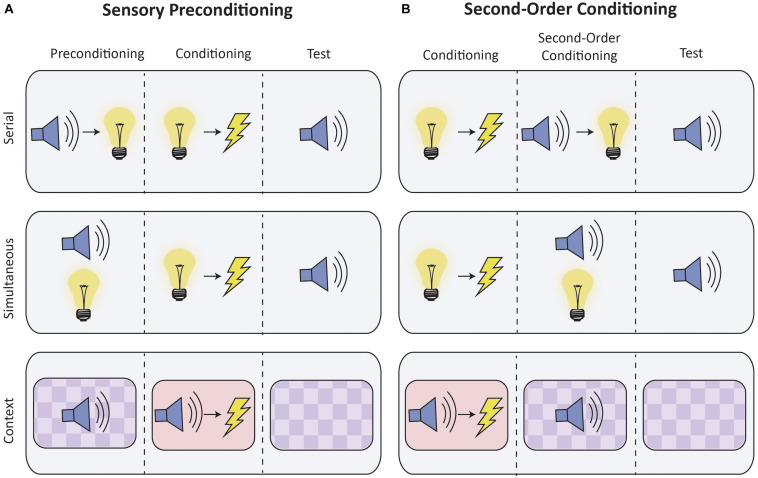
Schematic of higher-order conditioning procedures. The figure depicts typical experimental conditioning for sensory preconditioning **(A)** and second-order conditioning **(B)**, which are contrasted with control conditions (not shown). Discrete stimuli can be presented either serially (top row) or simultaneously (middle row). Higher-order conditioning of contextual stimuli is presented in the bottom row. Contexts are operationally defined as the static background stimuli provided by conditioning apparatus, and typically differ with respect to visual, tactile and olfactory characteristics. In the figure, contexts are distinguished by color and background.

Higher-order conditioning can be studied through two paradigms: sensory preconditioning and second-order conditioning (see Figure 1). In sensory preconditioning, two initially neutral stimuli (e.g., S2 and S1) are repeatedly presented together. One stimulus (S1) is then paired with the US. These phases are reversed during second-order conditioning: S1 is first directly paired with the US, after which it is then paired with S2. Importantly, in both sensory preconditioning and second-order conditioning, S2 acquires the ability to elicit a CR despite never being directly paired with the US. Through higher-order conditioning, both briefly presented discrete stimuli and static contextual stimuli can gain the ability to elicit responses (e.g., Rizely and Rescorla, 1972; Helmstetter and Fanselow, 1989; Iordanova et al., 2011; Robinson et al., 2018).

In the present article, we will consider the neuroanatomical basis of higher-order conditioning by selectively reviewing research that has examined the role of the retrosplenial cortex (RSC) in sensory preconditioning and second-order conditioning. For both forms of higher-order conditioning, we first briefly summarize the types of associations that may be formed and then we describe the putative role, if any, for the RSC. In addition, we identify gaps in the literature as well as avenues for future research. Finally, we consider the RSC’s role in higher-order conditioning with respect to other related structures.

### RSC Anatomy and Connectivity

**FIGURE 2:**
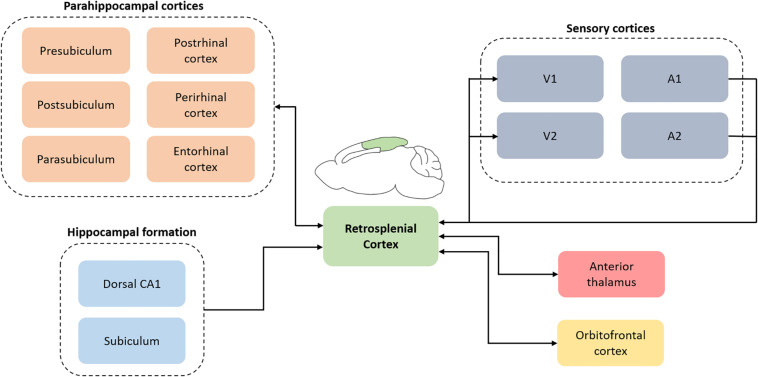
A simplified schematic depicting retrosplenial connections with cortical and subcortical regions. The connectomic diagram is centered around retrosplenial cortex and does not include the complex interactions between all regions. V1, primary visual cortex; V2, secondary visual cortex; A1, primary auditory cortex; A2, secondary auditory cortex.

The RSC (Brodmann area 29 and 30) was first described in humans but is evolutionarily conserved and is found in non-human primates and rodents (Vann et al., 2009). In rats, the RSC is located on the dorsomedial surface of the cerebrum and is cytoarchitecturally separated into dysgranular (Brodmann area 30) and granular RSC (Brodmann area 29). Connectomic studies using a combination of retrograde and anterograde tracers reveal extensive reciprocal connections of the RSC with multiple higher-order cortical structures including the hippocampal formation, parahippocampal region (e.g., perirhinal and postrhinal cortex) and the orbitofrontal cortex (see Figure 2; Van Groen and Wyss, 1990, 1992, 2003; Wyss and Van Groen, 1992; Miyashita and Rockland, 2007; Sugar et al., 2011). In addition, the RSC is well-connected with multiple sensory cortical areas; it receives inputs from auditory cortex and is reciprocally connected with the visual cortex (Vogt and Miller, 1983; Van Groen and Wyss, 1992, 2003; Todd et al., 2016b). The RSC also has reciprocal subcortical connections with several thalamic nuclei, the most prominent of which is the anterior thalamic nuclei (Sripanidkulchai and Wyss, 1986; Van Groen and Wyss, 1990, 1992, 2003). Functionally, the RSC contributes to several aspects of learning and memory, including spatial navigation, contextual and trace fear conditioning, and some aspects of Pavlovian and instrumental conditioning (see reviews by Vann et al., 2009; Bucci and Robinson, 2014; Miller et al., 2014; Mitchell et al., 2017; Corcoran et al., 2018; Todd et al., 2019). RSC pathology is also present in several disorders that include memory dysfunction, such as Alzheimer’s disease (Buckner et al., 2005) and PTSD (Sartory et al., 2013).

### RSC and Higher-Order Conditioning

#### Sensory Preconditioning

As previously noted, sensory preconditioning is an associative learning procedure in which a stimulus elicits a CR despite having never been directly paired with a US (Figure 1; Brogden, 1939). A key event in sensory preconditioning is thought to be the formation of stimulus-stimulus (S2–S1) associations that are acquired during the preconditioning phase when two neutral stimuli are presented together (Rizely and Rescorla, 1972; Rescorla and Cunningham, 1978). Importantly, this association is established prior to any presentation of a biologically significant US that will be later paired with S1 (e.g., Rizely and Rescorla, 1972; Rescorla, 1980). After a S2–S1 association is established, there are at least two ways by which S2 can gain the ability to elicit a CR (see Wong et al., 2019). One possibility is through an associative “chain,” such that S2 → S1 → US (Rizely and Rescorla, 1972). A second possibility is that during first-order conditioning of S1, the initial S2–S1 association allows for the retrieval of S2, which is then associated with the US (Holland, 1981).

Several studies have demonstrated that disruption of the RSC impairs sensory preconditioning in rats. For example, in an experiment by Robinson et al. (2011), rats first received either pre-training electrolytic or sham lesions of the RSC. During preconditioning, all rats received pairings of a discrete auditory stimulus followed immediately by a discrete visual stimulus, whereas a second auditory stimulus was presented alone. During first-order appetitive conditioning, the visual stimulus was then directly paired with a US (food pellets). Finally, responding to the auditory stimulus that was initially paired with the visual stimulus (“Paired”), and the auditory stimulus presented alone (“Unpaired”), was assessed in a test session in which no food was delivered. In this and all subsequent appetitive conditioning experiments, the response measured was the amount of time rats spent in the food cup during each stimulus presentation. Although sham rats demonstrated sensory preconditioning by responding more during the Paired vs. Unpaired stimulus, lesions of the RSC eliminated this effect. The finding was recently replicated and extended by Fournier et al. (2020), who demonstrated that pre-training neurotoxic or electrolytic lesions of the RSC prevent appetitive sensory preconditioning when auditory stimuli were used for *both* the first- and higher-order stimuli. Thus, the RSC appears to have an important role in forming associations both within and across sensory modalities.

The aforementioned studies utilized pre-training permanent lesions and therefore do not isolate a specific role for the RSC in sensory preconditioning. It is possible, for instance, that the RSC contributes to sensory preconditioning via either encoding or retrieval of S2–S1 associations, or both. However, an additional appetitive conditioning study by Robinson et al. (2014) demonstrated impaired sensory preconditioning when the RSC was temporarily inactivated (via chemogenetic methods) only during the preconditioning phase. This experiment therefore separated encoding from retrieval, by specifically targeting the RSC during preconditioning, and thus suggests an important role for the RSC in the initial encoding of neutral S2–S1 associations.

A recent experiment demonstrated a role for the RSC in higher-order conditioning using a version of sensory preconditioning that involved both discrete as well as static contextual stimuli and an aversive footshock US (Robinson et al., 2018). During preconditioning, rats were exposed to two contexts (A and B) that had distinct olfactory and visual characteristics. A tone stimulus was repeatedly presented in Context A, and a white noise stimulus in Context B. Thus, during preconditioning rats had the opportunity to associate each context with a specific auditory stimulus. During conditioning in a third context (C), one auditory stimulus was paired with shock and one was not. Finally, higher-order conditioning was assessed by measuring freezing behavior when rats were re-exposed to Contexts A and B in the absence of shock or auditory stimuli. Note that with this design, Contexts A and B were never directly paired with the shock. Instead, one context had been associated with an auditory stimulus that now predicted shock (“Paired” context) and the other context had been associated with an auditory stimulus that now predicted no shock (“Unpaired” context). Robinson et al. (2018) observed that control rats froze more in Paired vs. Unpaired context, however, rats with pre-training electrolytic lesions of the RSC froze equally in both contexts. One interpretation of these findings is that lesions of the RSC prevented the formation of associations between stimuli and the contexts in which they occurred.

#### Second-Order Conditioning

As a procedure, second-order conditioning is very similar to sensory preconditioning with the exception that the order of the initial two phases are reversed (see Figure 1). Thus, in second-order conditioning, S1 is first paired with the US, after which S2 is then paired with S1. The ability of S2 to elicit a CR can theoretically be mediated by one of several associations. For instance, S2 might elicit a CR due to an association between S2 and the *response* elicited by S1 (S–R), or an association between S2 and S1 (S–S). It is also possible that during the second phase, S1 evokes a representation of the US which is then associated with S2 (mediated conditioning). Which association occurs depends on how the stimuli are initially presented, as well as the overall experience with S1 (Rescorla, 1982). For example, sequential presentation of S2 and S1 appears to produce an S–R association, whereas simultaneous presentation results in an S–S association. In addition, Rescorla (1982) noted that extensive exposure to S1, either reinforced or non-reinforced, reduces S–S learning and permits S-R learning even when S2 and S1 were presented simultaneously.

To our knowledge, only one study to date has examined the role of the RSC in second-order conditioning (Todd et al., 2016a). In this conditioned suppression experiment, rats received either pre-training electrolytic lesions or sham lesions of the RSC. Next, both Sham and RSC-lesioned rats received first-order conditioning in which one visual stimulus was paired with shock (V1+), and one visual stimulus was presented alone (V2−). During first-order conditioning, both groups of rats first showed high levels of conditioned suppression to both V1+ and V2−, with Sham lesioned rats gradually reducing fear to V2−. However, RSC-lesioned rats were much slower to reduce fear to V2−, demonstrating a clear impact of the lesions on behavior. At the end of first-order conditioning, when both groups were successfully discriminating V1+ from V2−, each visual stimulus was then paired in a serial fashion with an auditory stimulus; V1+ was followed by A1 and V2− was followed by A2. Overall, there was greater conditioned responding to A1 than A2, and this did not differ between sham and RSC-lesioned rats. Thus, lesions of the RSC did not impair second-order conditioning. Todd et al. (2016a) suggested that the discrepancy between the involvement of the RSC in second-order conditioning and sensory preconditioning may be related to the type of association that is acquired. Indeed, in that experiment, the first- and second-order stimuli were presented serially, and subjects received an extensive amount of prior training with the first-order stimulus. As noted, both of these factors tend to promote S–R over S–S learning.

### Knowledge Gaps and Additional Considerations

Although the aforementioned experiments demonstrate a role of the RSC in sensory preconditioning with both discrete and contextual stimuli, several unanswered questions remain. For instance, no study to date has selectively inhibited RSC activity during either conditioning or testing of sensory preconditioning. Thus, although Robinson et al. (2014) demonstrated that the RSC is necessary for encoding of S–S associations, it is unknown if the RSC is also necessary for the retrieval, updating and/or reconsolidation of such associations. The role of the RSC in these phases might ultimately depend on the type of behavioral mechanism that is operating. One possibility is that RSC activity may be necessary during conditioning if, during S1–US pairings, S1 retrieves the representation of S2 such that S2 then undergoes mediated conditioning (Holland, 1981). An alternative possibility, which is not mutually exclusive from the first, is that RSC activity might be necessary during testing if the S2 → S1 → US chain is integrated during the final test phase. Interestingly, all prior discrete stimuli experiments have involved serial presentations of the higher- and first-order stimuli, which may involve chaining at the time of test (Sadacca et al., 2016; Sharpe et al., 2017; but see Wong et al., 2019).

In contrast to sensory preconditioning, there is currently no available data to support involvement of the RSC in second-order conditioning. However, before ruling out a role for the RSC completely, future experiments should examine if the RSC is involved in second-order conditioning with simultaneous presentation of S2 and S1, given that such presentation tends to promote S–S associations as in sensory preconditioning (Rescorla, 1982). These studies will be valuable in determining if the form of associations acquired (S–R or S–S) influence the recruitment of the RSC to second-order conditioning. In addition, such studies will provide valuable information about whether the RSC contributes to S–S associations when one stimulus is already associated with the US, or if the role of the RSC is specific to the encoding, storage, and/or retrieval of *neutral* S–S associations as in sensory preconditioning.

Apart from the types of associations that can be formed, other aspects of the procedure might impact whether or not the RSC is engaged during second-order conditioning. For instance, Holmes et al. (2018) demonstrated that a “dangerous” background context can impact where the brain stores S–S associations. When these associations are formed in a safe context, they involve the perirhinal cortex, but when they are formed in a dangerous context they rely on the amygdala. Critically, the presence of danger is typically a component of aversive second-order conditioning experiments, because the aversive US occurs during the first-order conditioning phase that by definition must precede the second-order phase. In contrast, this is often not the case in sensory preconditioning experiments, in which the US is typically not presented until the conditioning phase. Thus, the discrepancy in the contribution of the RSC to second-order conditioning and sensory preconditioning may be related to the valence of the context during the time that the higher-order associations are formed.

Finally, we note that the role of the RSC in sensory preconditioning is perhaps consistent with its role in other aspects of learning and memory, most notably contextual fear conditioning. Indeed, learning and memory for contexts is often thought to involve the integration of multiple sensory features in the environment (Fanselow, 2010), even in the absence of reinforcement, which is reminiscent of the task requirements inherent to sensory preconditioning. Further understanding of the role for the RSC in higher-order conditioning may thus inform the degree to which RSC function overlaps in these aspects of learning and memory.

### Roles of Related Cortical Regions

The experiments reviewed here demonstrate a role for the RSC in sensory preconditioning, specifically for the encoding of neutral S–S associations. Drawing from prior studies, it is possible to speculate how RSC function intersects with other circuits during preconditioning. For instance, inhibiting neural activity or protein synthesis in the perirhinal cortex (PER) following preconditioning reduces responding at test (Holmes et al., 2013; Wong et al., 2019). Further, inactivation of the orbitrofrontal cortex (OFC) during preconditioning also impairs responding to a preconditioned cue (Hart et al., 2020), and *in vivo* extracellular recordings indicate that OFC activity represents S–S associations acquired during preconditioning (Sadacca et al., 2018). Thus, the RSC, PER, and OFC may act in concert to facilitate the encoding of associations during preconditioning.

As described previously, S2–S1 associations encoded during preconditioning may allow S2 to be updated during conditioning of S1. This updating requires PER. For instance, blocking protein synthesis in PER immediately after conditioning impairs responding at test (Wong et al., 2019). It is possible that S–S associations encoded within the RSC are also updated during conditioning, although as noted, this has not been specifically tested. Nevertheless, it has been suggested that information encoded within the RSC might be updated through connections with the postrhinal cortex (POR; Bucci and Robinson, 2014); a suggestion that is supported by the putative role of POR in information processing that involves stimuli that undergo change (Ho and Burwell, 2014). Considering the direct anatomical projections between PER and POR (Furtak et al., 2007), it is possible that updating during conditioning might depend upon a distributed cortical network including PER, POR, and RSC.

A second form of integration we have described is one that occurs during the final test phase. In this case, initially encoded S–S associations are integrated with the conditioning memory as an associative chain to drive behavior (Sharpe et al., 2017). Inactivation of OFC during testing impairs responding to a preconditioned stimulus (Jones et al., 2012), suggesting that during testing the OFC is necessary for connecting associations acquired during the preconditioning and conditioning phases (Gardner and Schoenbaum, 2021). Although it is currently unknown if the RSC is also involved with integration at the time of test, such a role is perhaps consistent with the notion that the RSC is necessary when there is mismatch between previously acquired representations (Nelson et al., 2018). For example, although the S2–S1 association was initially encoded while both stimuli were neutral, during testing S2 now predicts S1 which has undergone a change in associative value. Future research is necessary to determine the role of the RSC during testing, and how it might contribute to a larger cortical network that supports higher-order conditioning.

## Conclusion

Here we examined the neural underpinnings of higher-order conditioning by reviewing the role of the RSC in sensory preconditioning and second-order conditioning. While several studies have demonstrated involvement of the RSC in sensory preconditioning, there is currently no evidence to suggest a role of the RSC in second-order conditioning. This apparent discrepancy may be related to several factors, including the type of associations formed in the two procedures (Todd et al., 2016a), or the status of the background context during the formation of higher-order associations (Holmes et al., 2018). Although there is a need to further examine the contributions made by the RSC to higher-order conditioning, especially second-order conditioning, the results from sensory preconditioning experiments indicate a role for the RSC in forming neutral stimulus-stimulus associations in the absence of reinforcement.

## Author Contributions

All authors contributed to the article and approved the submitted version.

## Conflict of Interest

The authors declare that this review was conducted in the absence of any commercial or financial relationships that could be construed as a potential conflict of interest.
